# 

**Published:** 1880-08

**Authors:** 


*CONTENTS.*

| | Page | | Page |
|---|---|---|---|
| Autumnal Diseases | 211 | The Best Summer Beverage | 227 |
| Washing | 213 | Households Contrasted | 228 |
| Feeding the Sick | 213 | Hair Washes | 230 |
| Cold in the Head | 215 | Pouting | 231 |
| Is Consumption Contageous ? | 216 | Corn Bread | 233 |
| Table Manners | 217 | Softening of the Brain | 234 |
| Tomatoes | 219 | Dieting for Health | 235 |
| Spitting Blood | 220 | Nursing Animosities | 236 |
| Small Bed Chambers | 221 | Suggestive | 236 |
| Each the Best | 222 | Finger Nails | 236 |
| Be Careful | 224 | Sudden Death | 237 |
| Eight to Sixteen | 225 | Wearing the Beard | 239 |
| Drinking Water | 226 | Eye-Lashes | 239 |
| Health is a Duty | 227 | Constipation | 240 |

